# Computer-assisted preoperative planning improves the learning curve of PFNA-II in the treatment of intertrochanteric femoral fractures

**DOI:** 10.1186/s12891-020-3048-4

**Published:** 2020-01-16

**Authors:** Dongdong Wang, Kun Zhang, Minfei Qiang, Xiaoyang Jia, Yanxi Chen

**Affiliations:** 1 Department of Orthopaedic Trauma, East Hospital, Tongji University School of Medicine, Shanghai, 200120 China; 2 Department of Orthopaedic Surgery, Zhongshan Hospital, Fudan University, 180 Fenglin Road, Shanghai, 200032 China

**Keywords:** Computer-assisted preoperative planning (CAPP), Learning curve, Intertrochanteric femoral fracture, Fracture fixation, Proximal femoral nail antirotation Asian version (PFNA-II)

## Abstract

**Background:**

Intertrochanteric femoral fractures are prevalent among the elderly, and usually demands surgical treatments. Proximal femoral nail antirotation Asian version (PFNA-II) is widely used for intertrochanteric fracture treatment. The computer-assisted preoperative planning (CAPP) system has the potential to reduce the difficulty of PFNA-II in the treatment of intertrochanteric fractures. The aim of the study was to investigate and compare the learning curves of PFNA-II treatment with CAPP and conventional preoperational planning methods for intertrochanteric femoral fractures.

**Methods:**

A total of 125 patients with intertrochanteric fracture who were treated with PFNA-II between March 2012 and June 2015 were retrospectively analyzed. Patients who underwent surgery with CAPP procedure by a junior surgeon were regarded as group A (*n* = 53); patients who underwent the conventional surgery by another junior surgeon were regarded as group B (*n* = 72). Each group was divided into three subgroups (case 1–20, case 21–40, case 41–53 or case 41–72).

**Results:**

The average operation time of group A was 45.00(42.00, 50.00) minutes, and in group B was 55.00 (50.00, 60.00) minutes (*P* < 0.01). Average radiation frequency and blood loss were 13.02 ± 2.32, 160.00 (140.00, 170.00) ml and 20.92 ± 3.27, 250.00 (195.00, 279.50) ml, respectively, with significant differences (*P* < 0.01). The learning curve of the surgical procedure in group A was steeper than that in group B. There were no significant differences in patient reported outcomes, hospital stay and complication rate between the two groups. Significant differences were observed between group A and B in Harris score at last follow-up in the AO/OTA type 31-A2 intertrochanteric fracture (*P* < 0.05).

**Conclusion:**

Compared with conventional preoperative planning methods, CAPP system significantly reduced operation time, radiation frequency and blood loss, thus reshaped the learning curve of PFNA-II treatment with lower learning difficulty.

**Trial registration:**

researchregistry4770. Registered 25 March 2019.

## Background

Intertrochanteric femoral fractures are the second most common type of hip fractures, and remain a major challenge for orthopaedic surgeons worldwide [1]. These fractures are usually classified as stable or unstable, with unstable fractures being more common [2]. Elderly people (age > 65 years) are particularly vulnerable to this type of injury owing to the high prevalence of osteoporosis or osteopenia in this population [3]. With the increase of the aging population, the incidence of geriatric intertrochanteric femoral fractures is also increasing. Because intertrochanteric femoral factures are associated with morbidity and mortality [4], and are usually followed by other physical ailments such as osteoporosis and diabetes [5, 6], these fractures continue to pose a considerable burden to the health-care system.

A previous study revealed that the primary cause of high mortality and complication rates in patients with an intertrochanteric femoral fracture is lack of exercise and particularly long-term bed rest [7]. Thus, aggressive and timely intervention is warranted. Surgical procedures play a major role in preventing bed-related complications and disability among the elderly [8]. The goals of surgery are to restore the anatomical alignment, maintain fracture reduction, and allow early rehabilitation [9]. Mobilization and full weight bearing as soon as possible are equally important after surgery [10].

To accomplish these goals, optimal implants with fixed stability and strength should be chosen. Various methods of fixation have been described for intertrochanteric fractures, including sliding hip screw, compression plating, fixed angle blade-plate, intramedullary nailing and external fixation. Intramedullary nailing has improved biomechanical features, and many surgeons would likely select intramedullary devices for the treatment of intertrochanteric femoral fractures [11]. The Proximal Femoral Nail Antirotation Asian version (PFNA-II), which was introduced in 2008 and modified to avoid lateral cortex impingement during the insertion of the nail [12], is now commonly used for pertrochanteric or intertrochanteric fractures in the geriatric population [13, 14].

To date, intramedullary fixation with PFNA-II has been considered a well-developed surgical technology in the treatment of intertrochanteric femoral fractures. Well-experienced surgeons could easily accomplish this surgery in < 30–45 min. However, it is still technically demanding with a definite learning curve, especially for beginners. A learning curve can be defined as an improvement in performance over time or with increasing experience or training. Understanding the average learning curve for surgery with a specific device is in the best interests of patient safety and is an important component of a surgeon’s learning process [15]. Poor reduction and failure of fixation in intramedullary fixation are closely related to insufficient assessment of the spatial characteristics of bone fragments and inappropriate selection of implants. Therefore, a precise and efficient preoperative design is particularly important, and it could enable lower the surgical difficulty of PFNA-II treatment [16].

A previous study has proved that an efficient and reliable system for computer-assisted preoperative planning (CAPP) provides excellent clinical outcomes in the treatment of humeral shaft fractures with locking plates [17]. Therefore, we presumed that the CAPP system could also provide a new approach with respect to preoperative design in PFNA-II treatment of intertrochanteric femoral fractures and lower the learning difficulty of this surgical procedure for beginners. To date, no such studies has investigated the impact of CAPP on the learning curve of PFNA-II treatment of intertrochanteric femoral fractures. The hypothesis of the current study is that the CAPP system might significantly reduce the learning difficulty of surgical treatments with PFNA-II in clinical practice.

## Methods

### Patient population

This retrospective study involved a single-center study of orthopedics at our hospital. Institutional review board approval (2019tjdx122) was obtained before collection of data. The medical records and imaging data of patients who underwent open reduction and intramedullary fixation with PFNA-II at our institution between March 2012 and June 2015 were retrospectively obtained and reviewed. Patients were included according to the following criteria: age ≥ 60 years, intertrochanteric femoral fracture, and injury-to-surgery interval within 1 week. Those who had pathological fractures, hip tumor, hip deformity, or other combined fractures were excluded.

A total of 125 patients were included in this study. For all patients, anteroposterior (AP) and lateral X-rays, computed tomography (CT) scans and three-dimensional (3D) reconstruction were performed preoperatively. And all CT images were obtained using a 16-detector spiral CT scanner (GE LightSpeed CT). During the same period, one junior surgeon performed surgeries in group A (*n* = 53) and another junior surgeon performed surgeries in group B (*n* = 72). The preoperative planning and evaluation in group A were conducted with the assistance of the CAPP system, whereas only conventional preoperative planning was conducted in group B. The two surgeons both had 4 years surgical experience and had the same medical background. In addition, both surgeons had undergone the same systematic training for 3 weeks under one senior surgeon before conducting operations independently. Further, the subsequent training of the two junior surgeons in the patient inclusion period was almost the same.

### Preoperative planning and evaluation

Preoperative planning and evaluation were usually conducted 1 or 2 days before surgery, to allow both the surgeons and the patients to prepare well and understand the surgery.

**Fig. 1:**
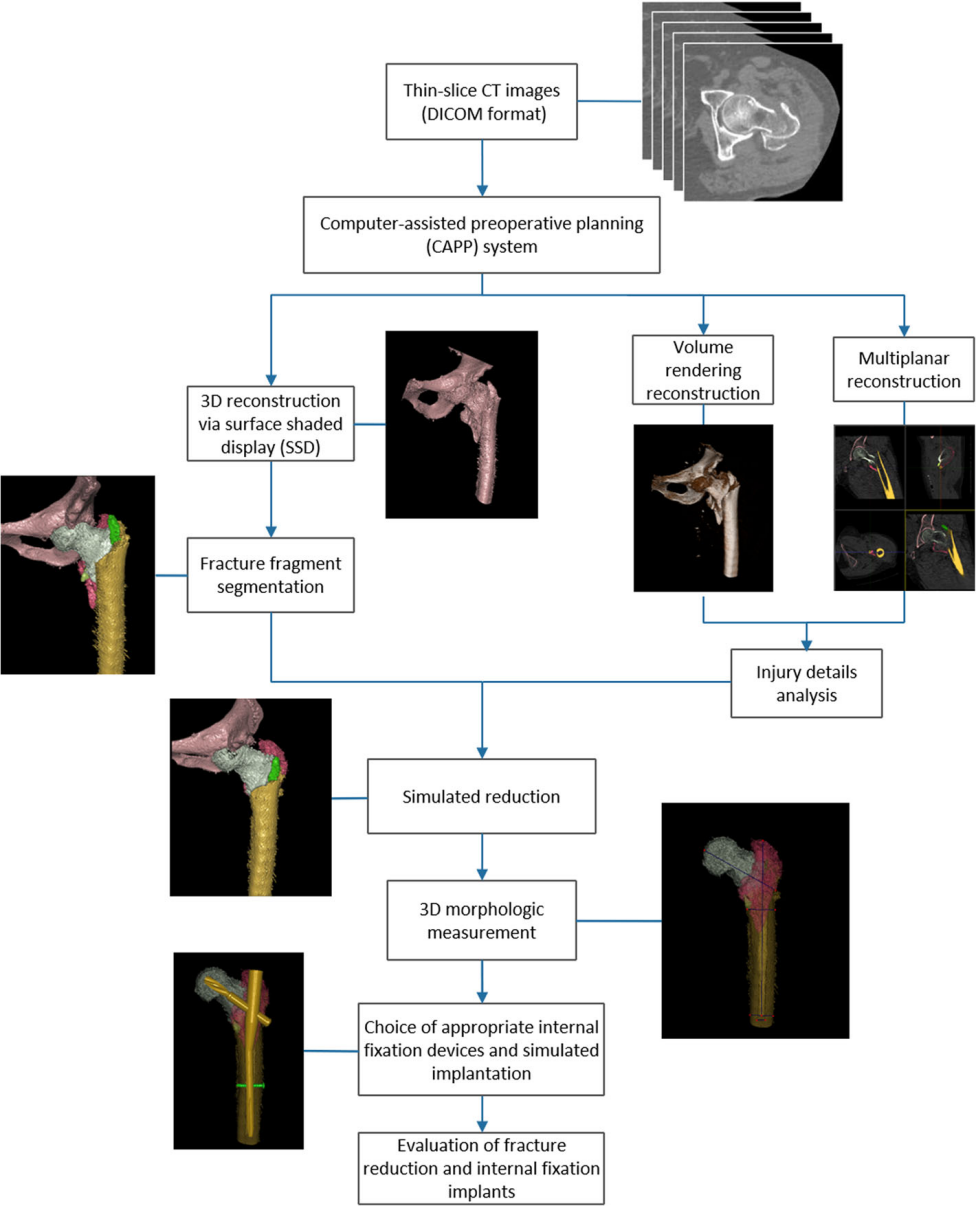
Flow diagram of computer-assisted preoperative planning for treatment of geriatric intertrochanteric fractures using PFNA-II

In group A, the original CT data of each patient were entered into the CAPP system (SuperImage Orthopedics Edition 1.0, Cybermed Ltd., Shanghai, China) [18].Thereafter, two-dimensional (2D) and 3D images of the fracture zone of the hip joint and surrounding structures were reconstructed through multiple planar reconstruction (MPR) and volume rendering (VR) technology, respectively. The CAPP procedures are shown in Fig. 1: (1) Fracture fragments segmentation using a surface shaded display (SSD) algorithm with a reconstruction interval of 0.625 mm. All fracture fragments were marked with different distinct colors. Injury details were analyzed in VR reconstruction and MPR mode. (2) Simulated fracture reduction using a semi-automatic fragment reconstruction approach on the 3D SSD images. (3) 3D morphological measurement of fracture fragments. The fracture fragments were measured three-dimensionally for choice of internal fixation devices. (4) Choice of appropriate internal fixation devices and simulated implantation. The type and length of the PFNA-II nails, helical blades and distal locking screws were measured accurately after simulated reduction, then simulated implantation using the measured internal fixation devices were conducted. (5) Evaluation of fracture reduction and internal fixation implants under a perspective mode. After CAPP procedures, fracture fragments segmentation time, simulated fracture reduction and fixation time, evaluation time as well as the total time were measured and recorded.

The preoperative planning in group B was based on radiographic and CT images (including 2D and 3D images) in combination with the surgeon’s experience, which is the typical preoperative planning method used by most orthopaedic surgeons.

### Surgical procedures

Before surgery, fracture reduction was performed with the aid of a traction frame. The operation was performed under general anesthesia. The patients were placed on the operating table in the supine position, with abduction of healthy lower limb and 10°–15° rotation of the hip joint on the affected side. Before surgery, fracture reduction was performed using a traction frame. Incision was made outside the hip joint after skin preparation, followed by insertion of a guide needle into center or slightly lateral area of the vertex of the greater trochanter. In the CAPP group, the choice among PFNA-II nails, helical blades, and distal locking screws was made according to the results of 3D morphological measurements. Therefore, appropriate internal fixation devices were inserted. However, in the conventional group, fracture reduction and suitable internal fixation were determined through preoperative planning and intraoperative fluoroscopy.

After surgery, anticoagulant drugs were administered to prevent deep vein thrombosis, and bisphosphonates were conventionally prescribed to elderly patients with osteoporosis. The patients were instructed to perform active contraction exercise for the quadriceps femoris muscle on the second day after the operation and gradually perform functional exercises on the bed. They were encouraged to sit up within 1 week, if tolerated, conduct off-bed non-weight-bearing activities with the assistance of a walking aid at 2–4 weeks after the operation; begin limited weight-bearing activities with walking aid assistance 4–6 weeks later; and complete weight-bearing activities without waling aid assistance after the clinical healing of the fracture. Postoperative radiographs were taken to confirm fracture healing.

### Outcome measurements

The operative time, radiation frequency, blood loss and fracture healing time of the two groups were analyzed and evaluated. Operative time was defined as the interval from skin insertion to the closure of the incision. It did not include the closed reduction time. Radiation frequency was defined as the number of times surgeon was exposed to intraoperative fluoroscopy during the operation. Patient-reported outcomes calculated as Harris hip scores were also compared. The Harris hip score was derived from the evaluation of pain, function, deformity, and range of movement of the hip joints. Harris hip scores were measured at 1 year after surgery. Postoperative complications were also recorded.

### Statistical analysis

Descriptive statistics, including means and standard deviations, median and interquartile ranges were calculated for demographic, operative, and outcome data (SPSS 22.0; IBM, Armonk, NY). Chi-square test was used to determine statistically significant differences in categorical variables. Student t-test and Mann-Whitney U test were used to determine statistically significant differences of continuous variables in clinical outcomes and patient reported outcomes. Analysis of Variance (ANOVA) test was used determine statistically significant differences of Harris scores among subtypes classified by fracture types. A *P* value < 0.05 or *P* value < 0.01 was considered significant. The learning curve was fitted with different curve estimation regression models (linear, logarithmic, quadratic, cubic, power) by SPSS 22.0, where y is the operative time and x is the chronological operation case number. The regression model of learning curve was finally set depending on the highest R value among the related plots and being consistent with the actual situation.

## Results

Basic characteristics including gender, age, fracture side, fracture type, comorbidities and hospital stay time of a total of 125 patients were demonstrated in Table 1. There exist no significant differences in gender, age, fracture sides, AO Foundation/Orthopaedic Trauma Association (AO/OTA) classification, comorbidities and hospital stay time.

**Table 1:** Baseline characteristics

	Group A(*n* = 53)	Group B(*n* = 72)	*P* value
Gender			0.907
Male	23(43.40%)	32(44.44%)	
Female	30(56.60%)	40(55.56%)	
Age (y)	75.26 ± 6.65	76.83 ± 6.16	0.176
Fracture side			0.110
Left	24(45.28%)	43(59.72%)	
Right	29(54.72%)	29(40.28%)	
Evans classification			0.910
I b	12(22.64%)	18(24.32%)	
I c	9(16.98%)	15(20.27%)	
I d	15(28.30%)	19(26.39%)	
II	17(32.08%)	20(27.78%)	
AO/OTA classification			0.867
31-A1	12(22.64%)	18(24.32%)	
31-A2	24(45.28%)	34(47.22%)	
31-A3	17(32.08%)	20(27.78%)	
Comorbidities			
Osteoporosis	45(84.91%)	58(80.56%)	
Cardiovascular and cerebrovascular diseases	20(34.74%)	33(45.83%)	
Respiratory disease	8(15.09%)	12(16.67%)	
Diabetes mellitus	15(28.30%)	18(25.00%)	
Hospital stay (d)	14.00 (2.00)	14.00 (1.00)	0.062

Unless otherwise noted, data are means±SD or median (interquartile range), numbers of subjects and percentages in parentheses

**Fig. 2:**
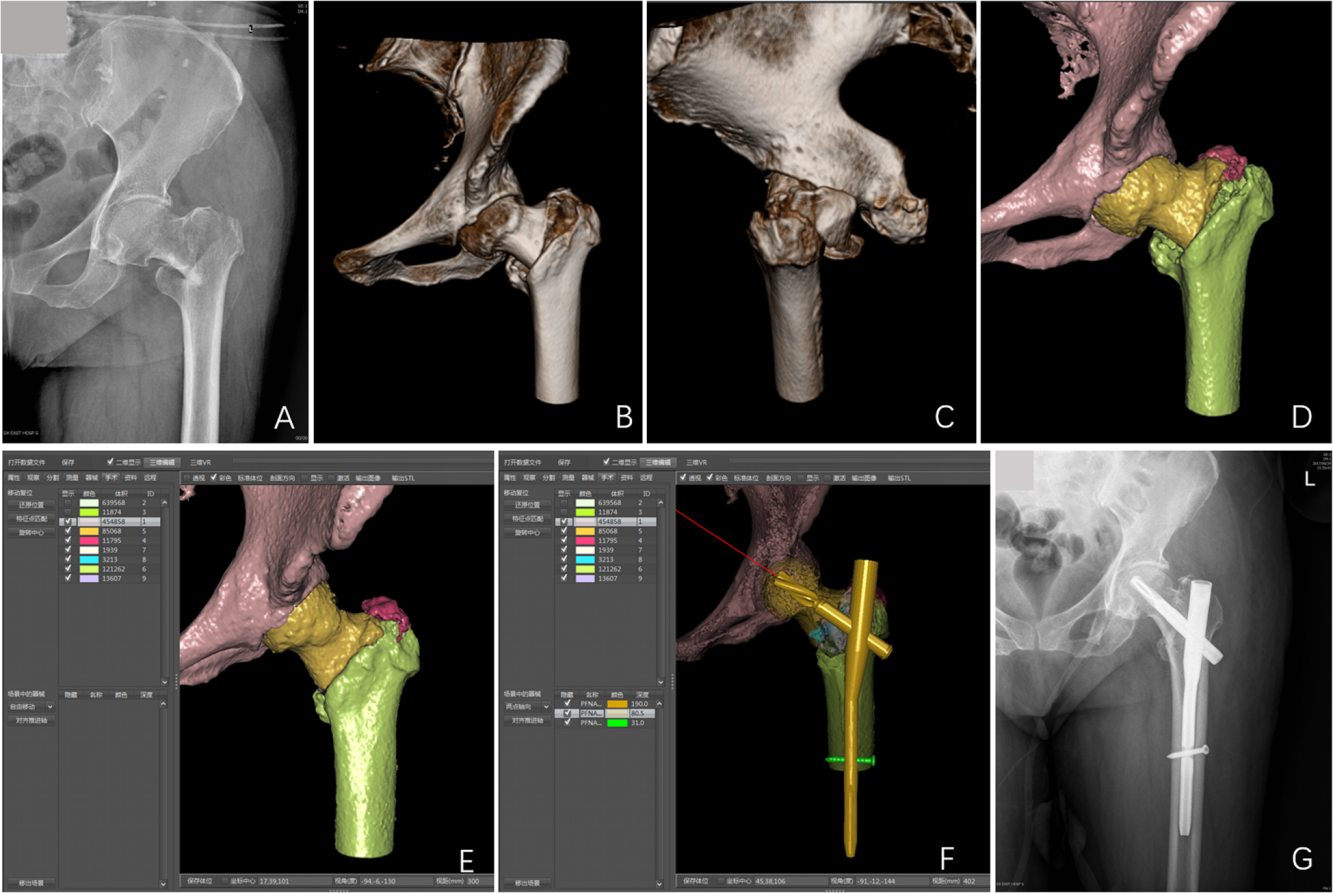
The computer assisted pre-operative planning for the treatment of intertrochanteric fracture. A 75-year-old female complained of left hip pain and movement disorder due to tumble. **a** Preoperative anterior-posterior (AP) X-ray showing left intertrochanteric fracture (AO/OTA 31-A2); **b**, **c** 3D volume rendering images revealed comminuted intertrochanteric fracture with one intermediate fragment; **d** The shaded surface display image of the fracture fragment was labeled by 3D interactive and automatic segmentation; **e** Simulated fracture reduction; **f** The fracture with appropriate internal fixation devices was simulated; **g** Postoperative AP X-ray of 12 months after surgery showing good reduction of fracture fragments and bone healing

In this study, the operation procedures were simulated and evaluated using the CAPP system, and almost all preoperative designs were successfully executed intraoperatively (Fig. 2). The actual type of internal fixation devices, type and length of the screw blade, type and length of the main nail, and length of the distal locking screw applied during the operation remained the same as the simulated choice of internal fixation devices in CAPP system. None of the patients in the CAPP group underwent repeated adjustments and replacement of the implants, which reduced the operation time and radiation exposure of the surgeons.

**Fig. 3:**
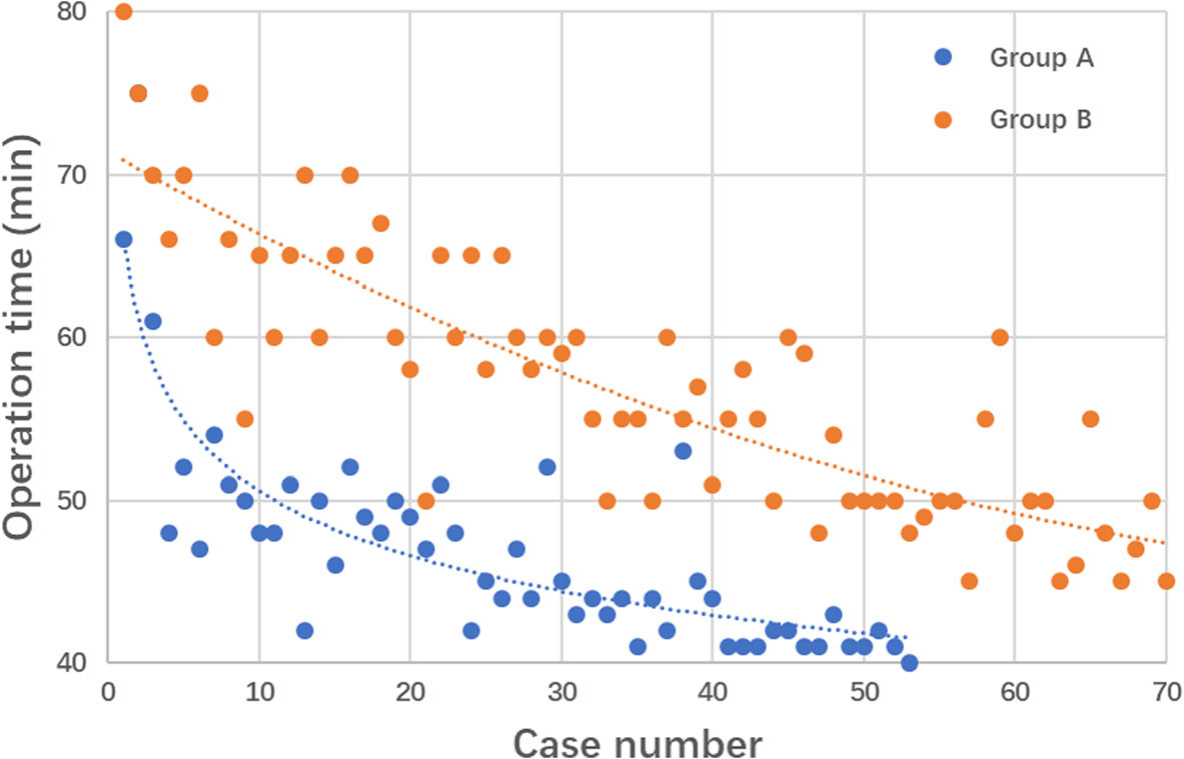
Learning curves of surgical treatments with PFNA-II in CAPP and the conventional groups. Group A: y = 66.341x^-0.118^, *R*^2^ = 0.701; Group B: y = 0.0027 × ^2^–0.5334x + 71.401, *R*^2^ = 0.733

The time of segmentation, stimulated reduction and fixation, evaluation and total CAPP time of group A are summarized in Table 2. ANOVA test revealed no significant differences in segmentation time (*P* = 0.954), stimulated reduction and implantation time (*P* = 0.776), evaluation time (*P* = 0.574) and total CAPP time (*P* = 0.530) among subgroups (1–20, 20–41 and 41–53/41–72). The operation time of group A and B were also demonstrated. There were significant differences among the subgroups in group A (*P* < 0.01) and group B (*P* < 0.01). Moreover, significant differences existed in operation time among subgroups between group A and B (*P* < 0.01). Figure 3 shows the learning curves of the two groups. Group A: y = 66.341x^-0.118^, *R*^2^ = 0.701, power regression; Group B: y = 0.0027 × ^2^–0.5334x + 71.401, *R*^2^ = 0.733, quadratic regression. The learning curve of the surgical procedure in group A was steeper, and was obviously below that of group B.

**Table 2:** CAPP and operation time

	1–20	21–40	41–53/41–72	Total
CAPP time (min)				
Segmentation	6.50 (5.25, 8.00)	6.00 (5.00, 8.75)	6.47 (4.75, 8.50)	6.89 ± 2.55
Simulation of reduction and implantation	13.85 ± 3.34	13.10 ± 3.26	13.52 ± 3.30	13.49 ± 3.25
Evaluation	5.00 (2.25, 7.00)	4.00 (2.00, 6.00)	4.50 (2.75, 5.25)	4.50 (2.00, 6.00)
Total time	25.50 (21.00, 28.00)	24.00 (22.00, 26.00)	24.00 (22.50, 26.25)	24.73 ± 4.01
Operation time (min)				
Group A	50.00 (48.00, 52.75)	44.00 (43.25, 47.00)	41.00 (41.00, 42.00)	45.00 (42.00, 50.00)
Group B	65.50 (60.00, 70.00) ^*^	58.00 (55.00, 60.00) ^*^	50.00 (47.25, 54.75) ^*^	55.00 (50.00, 60.00) ^*^

Data are median (25% quartile, 75% quartile) or mean ± SD

*P < 0.01, significant difference between the two groups

Clinical outcomes including operation time, radiation frequency and blood loss of group A and B are shown in Table 3. The average operation time of group A and B was 45.00 (42.00, 50.00) and 55.00 (50.00, 60.00) min, respectively. The average radiation frequency of each group was 13.02 ± 2.32 and 20.92 ± 3.27 times. The average blood loss of the two groups was 160.0 (140.0, 170.0) and 250.0 (195.0, 279.5) mL. Significant differences (*P* < 0.01) were observed between group A and group B in operation time, radiation frequency and blood loss. Three patients in group B had separation and displacement of the trochanteric bone fragments, whereas no separation or displacement of fractures was observed in group A. The observed complications are also demonstrated in Table 3. In group A, one patient complicated with Alzheimer’s disease developed wound infection due to repeated scratching, and the wound finally healed after 2 weeks of outpatient dressing changes. One patient developed cerebral infarction in the left temporal and occipital area, with decreased sensation and muscle strength in the contralateral limb at 1 week after surgery. Two patients died 8 weeks after surgery because of heart failure combined with pulmonary infection. The complication rate in group A was 7.55% (4/53). While in group B, there were 3 cases of urinary-tract infection, 4 cases of death (1 duo to pulmonary infection, 1 due to renal failure, and 2 due to cerebral infraction combined with respiratory failure), with a complication rate of 9.72% (7/72). There was no significant difference in complication rate (*P* > 0.05).

**Table 3:** Clinical outcomes

	Group A (*n* = 53)	Group B (*n* = 72)	*P* value
Operation time (min)	45.00(42.00, 50.00)	55.00 (50.00, 60.00)	< 0.01^*^
Blood loss (mL)	160.00 (140.00, 170.00)	250.00 (195.00, 279.50)	< 0.01^*^
Radiation frequency (times)	13.02 ± 2.32	20.92 ± 3.27	< 0.01^*^
Postoperative complications			
Wound infection	1	0	
Urinary-tract infection	0	3	
Cerebral infraction	1	0	
Death	2	4	
Complication rate (%)	7.55% (4/53)	9.72% (7/72)	0.671

Data are median (25% quartile, 75% quartile) or mean ± SD

*significant difference (*P* < 0.01)

As demonstrated in Table 4, the average follow-up time was 18.00 (15.00, 21.00) months and 19.00 (18.00, 21.00) months, respectively. The average fracture healing time was 18.00 (15.50, 19.00) weeks and 18.00 (16.00, 20.00) weeks, respectively. No statistically significant difference in follow-up time and fracture healing time were observed (*P* > 0.05). Most patients in each group achieved good postoperative recovery and could walk without crutches by 4 months after surgery. The Harris scores of patients at the last follow-up were evaluated and analyzed. Group A had an average Harris score of 88.67 ± 2.50, including 15 excellent cases, 27 good cases and 3 fair cases. Group B had an average Harris score of 87.52 ± 3.18, with 12 excellent cases, 31 good cases and 5 fair cases. There were no significant differences in AO/OTA type 31-A1 and 31-A3 intertrochanteric fractures (*P* > 0.05); however, significant differences were observed in AO/OTA type 31-A2 intertrochanteric fracture (*P* < 0.05).

**Table 4:** Patients reported outcomes

	Group A (*n* = 45)	Group B (*n* = 48)	*P* value
Follow-up time (mon)	18.00 (15.00, 21.00)	19.00 (18.00, 21.00)	0.229
Fracture healing time(w)	18.00 (15.50, 19.00)	18.00 (16.00, 20.00)	0.317
Harris score			
31-A1	86.67 ± 2.50	88.83 ± 3.95	0.167
31-A2	89.57 ± 2.65	86.82 ± 2.83	0.010^*^
31-A3	88.29 ± 3.92	87.63 ± 2.53	0.512
total	88.64 ± 2.79	87.60 ± 3.02	0.088

Data are mean ± SD or median (25% quartile, 75% quartile)

*significant difference (*P* < 0.05)

## Discussion

Intertrochanteric femoral fracture is prevalent among the elderly population, and usually requires surgical treatments. To date, the ideal implant for the treatment of intertrochanteric fractures remains controversial [19]. PFNA-II has been considered a well-developed surgical technology in the treatment of intertrochanteric femoral fractures [13, 20]. Although this kind of surgery is relatively easy to accomplish for experienced surgeons, it is still challenging for beginners. When performed by young surgeons, operative failure and loss of self-confidence may occur because of insufficient assessment of bone fragments and inappropriate selection of implants. Therefore, accurate preoperative design at the early learning stage is particularly important. Because it could help lower the learning difficulty and increase self-confidence in beginners. This study was performed to validated whether CAPP could help reduce the learning difficulty of PFNA-II treatment of intertrochanteric femoral fractures of beginners.

In conventional preoperative planning, the 3D morphological characteristics of fractures are imagined by surgeons based on 2D images (radiographs, CT scans or magnetic resonance imaging (MRI) slices). This kind of preoperative planning relies on the clinical experience and subjective imagination of surgeons, which may be distorted and inaccurate. Many studies investigated novel preoperative planning methods in order to improve the efficiency and effectiveness. One of them was 3D printing technology. A 3D printing model can provide a direct and interactive display of fracture characteristics, and can be used to perform virtual procedures in vitro such as fracture reduction and simulation surgery [21]. However, this technology is available for only a few patients because it is expensive and time consuming, which limited its clinical applications. Okada et al. [22] described a method that extract fracture lines based on 3D curvature analysis. It was used for the repositioning and registration of bone fragments in proximal femoral fractures. This provided a new model of preoperative planning for computer-guided fracture reduction.

Our CAPP system could show the fracture characteristics of patients and pre-perform surgical procedures (including fracture reduction and implantation simulation) in a direct and virtual manner. Moreover, the system enables 3D morphological measurements of the femoral head, shaft, and diameter of femur after reduction, which could directly guide the selection of implants. The average CAPP time was < 1 h, which was not time-consuming. A previous study [23] has also demonstrated that CAPP based on virtual surgical technology has several advantages, including shorter preoperative planning time, shorter interval from injury to surgery, and shorter duration of hospital stay, in the treatment of proximal humeral fractures.

CT/3D CT data are necessary for reconstruction and assessment in the CAPP system. Plain radiographs have some limitations; for example, coronal fragments are difficult to identify on radiographs [24]. However, knowing the incidence and morphology of coronal fragments are important to avoid potential intraoperative pitfalls. Although plain radiographs may reveal fracture details with a relatively lower cost for patients, CT/3D CT examination was more reliable and helpful for preoperative assessment, especially for intramedullary fixation [25].

Learning curves are important for surgeons to understand and master a certain surgery with a specific device. Our hypothesis was that surgical experience has significant effects on the operative time. The results showed that with the accumulation of surgical experience, the operation time gradually decreased and the learning curve for intramedullary fixation with PNFA-II became acceptable. As shown in Fig. 3, the learning curve of CAPP group was steeper than that of the conventional group, indicating that the surgical procedure could be managed faster with less surgical volume with the assistance of CAPP. Such conclusions were consistent with those of other similar learning curve studies [26, 27]. The average operation time with a learning curve effect was about 40 min, consistent with other previous studies [20].

Observing and performing operations are important parts of surgical training in the orthopedic field [28]. PFNA treatment is a highly standardized procedure, and is often used for training younger surgeons. The common way of training is to allow residents to perform operative procedures under the supervision of senior surgeons. Cases involving patients with morbidity, osteoporosis and the trauma setting are often challenging problems for beginners. Thus, a direct and virtual way for simulating surgical procedures has great value. Previous studies revealed an increasingly important role of surgical simulators [29, 30] as well as virtual reality assistants [31] in the surgical training of orthopaedic resident. Although the CAPP system in this study only served as a useful tool for preoperative design, we believe that it might have the potential to become a routine part of resident education in orthopaedic surgery. Residents could learn surgical procedures by observing senior surgeons, performing surgical simulation with CAPP, and conducting actual operations step by step. Further studies investigating the organic integration of CAPP and virtual reality display could guide the path for more efficient and precise preoperative designs.

Moreover, the application of CAPP enables preventing lateral femoral wall fracture during the operation. The lateral femoral wall, firstly introduced by Gotfried [32], has gain its recognition by orthopaedic surgeons in the treatment of intertrochanteric fractures. Im et al. [33] used dynamic hip screws (DHS) to treat 66 patients with intertrochanteric fractures classified as AO/OTA type 31-A1. Nine patients had comminution of the lateral cortex either during the operation or postoperatively. Palm et al. [34] reported about the integrity of the lateral femoral wall as a main predictor of reoperation. Hsu et al. [35] demonstrated that lateral femoral wall thickness is a reliable predictor of postoperative lateral wall fracture in intertrochanteric fractures. And intertrochanteric fractures with a lateral wall thickness < 20.5 mm should not be treated with DHS alone. The intact lateral wall has played a key role in preoperative reduction, implants selection and postoperative stability of internal fixation in unstable intertrochanteric fractures. Therefore, maintaining the integrity of the lateral wall should be an important objective in all stabilization procedures for unstable trochanteric fractures. Compared with the conventional preoperative planning, CAPP has shown excellent effects in terms of maintaining the integrity of the lateral femoral wall during the preoperative designs processes. In the CAPP group, there were no lateral wall fractures during the operation and postoperatively.

Patients in the CAPP and conventional group all performed functional exercises after surgery. The Harris score at 1-year follow-up showed satisfactory clinical effects (88.64 ± 2.79 and 87.60 ± 3.02 in the CAPP and conventional groups, respectively). Especially, the Harris score in AO/OTA type 31-A2 intertrochanteric fractures of the CAPP group was significantly higher than that in the conventional group. CAPP has the potential to improve the clinical outcomes of PFNA-II treatment of intertrochanteric fractures, especially in comminuted fractures with separated greater and lesser trochanters, and varus deformity. However, according to the AO/OTA-2018 classification, type 31-A2 fracture (trochanteric region fracture) are defined as multi-fragmentary per-trochanteric lateral wall incompetent (≤ 20.5 mm) fracture [36]. This might lead to bias because the type 31-A2 fracture in AO/OTA-2007is totally different from that in the new classification. AO/OTA-2018 stressed the importance of the lateral wall by using the involvement of lateral wall as classification basis not the lesser trochanter fragments.

Previous studies have demonstrated that extended and progressive exercise can significantly promote the rehabilitation of limb function and improve the quality of life [37]. Postoperative rehabilitation exercise in elderly patients are important for the limb function recovery. In addition, postoperative complications showed no significant differences between the two groups. The complication rates were similar with those reported in previous studies [20, 38].

This study has some limitations. First, many factors could influence the operation time, including the complexity of fractures. Because complicated fracture would increase the operation time, surgeons at the early stages of the learning curve usually choose relatively simple and typical cases, which is conducive to mastering the surgical technique and improving self-confidence. This may lead to bias because complex cases are rare. Randomized studies are needed to reduce the selection bias. However, as this was a retrospective study, the bias was difficult to eliminate. This will inevitably affect the evaluation of the learning curve. Second, potential bias due to difference in the skill level of surgeons could exist, because we could not expect the two surgeons to be “identical clones”. However, this was an inherent limitation of this kind of study to investigate an impact on the learning curves regardless of prospective or not, like investigating the impact of training level on the learning [39]. Considering the non-divergent characteristics of included patients, as well as the same medical background and surgical exposure of the two surgeons, we still believe that the current study is qualified to clarify the topic. Third, the follow-up evaluations were performed in the outpatient setting and, some patients in both groups were lost to follow-up. This may have affected the evaluation of the postoperative patient-reported outcomes. In addition, the follow-up time is not sufficient for the long-term evaluation of function. Lastly, the CAPP system is not popular, and our results could only represent the improvement of clinical skills at our institution. Potential bias may also exist in this regard. Further multi-center prospective and randomized studies are needed to evaluate the importance of CAPP in improving the learning curves of PFNA-II treatment of intertrochanteric fracture patients.

## Conclusion

This study indicated that compared with the conventional preoperative planning, CAPP significantly reduced the operation time, radiation frequency and blood loss of PFNA-II intramedullary fixation for femoral intertrochanteric fractures. The preoperative designs generated by the CAPP system reshaped the learning curve of PFNA-II treatment with significantly lower learning difficulty. Professional training to master this surgical technique is essential for junior surgeons. When performed by surgeons having a mastery of the technique, PFNA-II treatment could be a reliable and effective option for patients with intertrochanteric femoral fractures.

## Data Availability

The datasets used and/or analyzed during the current study are available from the corresponding author on reasonable request.

## Abbreviations

2D: Two-dimensional
3D: Three-dimensional
AO/OTA: AO Foundation/Orthopaedic Trauma Association
AVOVA: Analysis of Variance
CAPP: Computer-assisted preoperative planning
CT: Computed tomography
DHS: Dynamic hip screw
MPR: Multiple planar reconstruction
MRI: Magnetic Resonance Imaging
PFNA-II: Proximal femoral nail antirotation Asian version
SSD: Surface shaded display
VR: Volume rendering
